# Publication Pressure and Burn Out among Dutch Medical Professors: A Nationwide Survey

**DOI:** 10.1371/journal.pone.0073381

**Published:** 2013-09-04

**Authors:** Joeri K. Tijdink, Anton C. M. Vergouwen, Yvo M. Smulders

**Affiliations:** 1 VU University Medical Centre, Department of Internal Medicine, Amsterdam, The Netherlands; 2 Tergooi Hospital, Department of Psychiatry, Blaricum, The Netherlands; 3 Saint Lucas Andreas Hospital, Department of Psychiatry, Amsterdam, The Netherlands; Consejo Superior de Investigaciones Cientifics, Spain

## Abstract

**Background:**

Publication of scientific research papers is important for professionals working in academic medical centres. Quantitative measures of scientific output determine status and prestige, and serve to rank universities as well as individuals. The pressure to generate maximum scientific output is high, and quantitative aspects may tend to dominate over qualitative ones. How this pressure influences professionals’ perception of science and their personal well-being is unknown.

**Methods and Findings:**

We performed an online survey inviting all medical professors (n = 1206) of the 8 academic medical centres in The Netherlands to participate. They were asked to fill out 2 questionnaires; a validated Publication Pressure Questionnaire and the Maslach Burnout Inventory. In total, 437 professors completed the questionnaires. among them, 54% judge that publication pressure ‘has become excessive’, 39% believe that publication pressure ‘affects the credibility of medical research’ and 26% judge that publication pressure has a ‘sickening effect on medical science’. The burn out questionnaire indicates that 24% of medical professors have signs of burn out. The number of years of professorship was significantly related with experiencing less publication pressure. Significant and strong associations between burn out symptoms and the level of perceived publication pressure were found. The main limitation is the possibility of response bias.

**Conclusion:**

A substantial proportion of medical professors believe that publication pressure has become excessive, and have a cynical view on the validity of medical science. These perceptions are statistically correlated to burn out symptoms. Further research should address the effects of publication pressure in more detail and identify alternative ways to stimulate the quality of medical science.

## Background

Publication of scientific research papers is important for medical professionals, particularly in academic environments. Scientific output is used to rank prestige and status of both academic medical centres as well as of individual medical staff [1]. Quantitative measures of scientific performance, such as the Hirsh index [2], have become particularly important, as these directly influence grant proposals, financial rewards and career potential [3]–[8].

Parallel to increased emphasis on scientific output measures, the quantity of (medical) scientific output has increased enormously in the past decades: the number of scientific journals increased from 5000 in 1997 tot 8000 journals in 2010 as registered by the ISI Web of Knowledge. (ISI Web of Knowledge, http://www.webofknowledge.com, consulted March 2012), and the amount of scientific papers doubles every 12 years (http://www.scopus.com).

The increasing emphasis of scientific performance potentially raises pressure on medical professionals to publish, and may intensify competition between them. This competition for papers and funding is often considered a salutary driving force among scientists, increasing efficiency and productivity [9]. Potential negative effects of a competitive publication culture with a focus on quantitative performance are not often considered. Concerns have however been expressed that scientists are continuously producing ‘publishable’ results at the expense of quality, validity, scientific rigour and personal integrity [10], and published negative research results have decreased over the years [11]. Consequently, clinical practice based on research outcomes may be jeopardised [12], [13].

Excessive emphasis on scientific output may also affect academic activities that compete with science for time and attention, such as clinical and educational activities [14], as these activities can be perceived disadvantageous and less important while affected by publication pressure [13], [15].

Finally, it is conceivable that mental well-being benefits from working in an environment with a healthy scientific culture. In this respect, increasing evidence that burn out symptoms are common among medical doctors and residents [16]–[20] is noteworthy. Burn-out symptoms may impact on academic tasks (ie not just science, but also patient care and education) and has previously been suggested to be related to publication pressure [13].

Many of the aforementioned phenomena are difficult to address in quantitative terms and epidemiological studies. However, important information can be obtained from anonymous questionnaires in academic professionals. The aim of our study is therefore to assess the perception of publication pressure among a large group of medical professionals in an advanced stage of their academic career. We also investigated how publication pressure relates to their view on the quality of medical science, as well as to aspects of their personal well-being.

## Methods

### Procedure and Participants

Full professors working at one of the 8 academic medical centres (AMC’s) in The Netherlands were sent an invitational e-mail in September 2011.

The deans of 4 AMC’s provided the e-mail addresses of their professors (n = 600) to the research team. The other 4 AMC’s chose to distribute the electronic link to the questionnaire internally.

The invitational e-mail explained the objectives of the study, using neutral terms as 'work engagement' and 'publication culture', and provided them with a link to an anonymous online questionnaire on a protected website. Those who not responded were sent a reminder after 3 weeks.

### Variables

The questionnaire contained, apart from demographic data, 2 parts; a specifically designed publication pressure questionnaire and a validated burn out questionnaire.

The Publication Pressure Questionnaire (PPQ) contained 24 statements (table 1), the responses to which were scored on a 5-point Likert scale. The questionnaire was initially divided into 3 broad domains by intuition:

**Table 1:** Publication pressure questionnaire (PPQ).

Questions (Domain)	Likert Scale Score (SD)	% agreement(4–5 or 1–2* on Likert Scale)
***1. Without publication pressure, my scientific output would be of higher quality (PP1)***	**2,6 (1,2)**	**23%**
*2. Publishing scientific articles is the most important part of my professorship (PP1)*	3,2 (1,1)	41%
***3. My scientific publications contribute to better (future) medical care*** * ***(PP1)***	**2,2 (0,8)**	**74%**
*4. The number of scientific publications determines my status/prestige (PP3)*	4,0 (0,8)	79%
*5. The number of scientific publications of my colleagues determines their status/prestige (PP2, PP3)*	4,0 (0,8)	82%
***6. I experience judgement of my publications by colleagues as stressful (PP1, PP3)***	**2,4 (1,1)**	**18%**
***7. I experience the scientific output criteria set by the university for my appointment and reappointment*** ***as stimulating*** * ***(PP1, PP3)***	**3,2 (1,1)**	**24%**
***8. Publication pressure puts pressure on my relations with fellow researchers (PP1,PP2)***	**2,8 (1,2)**	**32%**
*9. Publication pressure increases my scientific output, without loss of quality* * *(PP1)*	3,5 (1,0)	15%
***10. I suspect that in some colleagues publication pressure leads to (un)intentional data manipulation (PP2)***	**3,0 (1,1)**	**33%**
***11. Worldwide, publication pressure adds validity to medical science*** * ***(PP2)***	**3,7 (1,0)**	**11%**
***12. On a global scale, publication pressure causes serious doubts regarding the validity of research*** ***results (PP2)***	**3,1 (1,1)**	**38%**
***13. I think the pressure to publish has become excessive (PP1, PP2))***	**3,4 (1,2)**	**54%**
*14. Fellow medical experts envy me because of my professorship (PP3)*	3,0 (0,9)	13%
***15. The competitive scientific culture stimulates me to publish more*** * ***(PP1)***	**3,0 (1,0)**	**40%**
*16. I experience my professorship as a burden and I sometimes long back to when I was not in this position (PP3)*	2,0 (1,1)	13%
***17. My colleagues mainly judge me on my publication record (PP1, PP3)***	**2,6 (1,1)**	**22%**
*18. Despite the pressure to publish, I enjoy investing in other activities which come with my professorship* * *(PP1)*	1,9 (0,8)	85%
***19. Fellow professors adequately maintain their clinical and educational skills, despite publication pressure*** * ***(PP2)***	**2,9 (0,9)**	**36%**
*20. Team spirit and collegiality are, in my hospital, key aspects in all professors’ appointment procedures* * *(PP3)*	3,0 (1,1)	34%
***21. I cannot trust my colleagues on innovative research proposals (PP1, PP3)***	**2,1 (1,0)**	**11%**
*22. I am too much involved in management (PP3)*	3,2 (1,2)	43%
*23. Professorship is difficult to combine with training and educating residents (PP3)*	2,9 (1,0)	25%
***24. The urge to publish makes science sick (PP2)***	**2,6 (1,2)**	**25%**

In **bold** the statements who are part of the validated questionnaire.

* inversed questions; higher scores for disagreement.

1. pressure to publish personally experienced by the respondent
2. publication pressure in general terms in the academic work place, as perceived by the respondent
3. publication pressure relating to the scientist’s position and status (e.g. promotion, re-appointment, etc)

After statistical validation of the PPQ with Confirmatory Factor Analysis, Explanatory Factor Analysis and Item Response Theory, we condensed the questionnaire to 14 items, having publication pressure as a single factor [21].

The Likert scale scores were assigned 1 to 5 points such that higher scores reflected higher pressure. We labelled different statements as positive and negative to avoid 'yeah-saying' [22]. Negative statements were scored inversely (ie totally disagree = 5 points instead of 1 point, see table 1).

Burn out was measured using the Dutch version [23] of the Maslach Burnout Inventory (MBI) Human Services Survey [24], which is designed specifically for use in people working in human services and health care. We chose the MBI since it is the primary measurement for work-related mental status in otherwise healthy people. Also, the MBI is most frequently used in similar types of scientific research [16]–[20].

The Dutch version of the MBI consists of 20 items covering the three domains of burn out: emotional exhaustion (EE, 8 items, key symptom), depersonalisation (DP, 5 items) and personal accomplishment (PA, 7 items). Emotional exhaustion (EE) is characterized by loss of energy at work and a negative attitude towards work-related activities. Depersonalisation (DP) relates to a kind of alienation from work, where interest in job and colleagues is lost.

Personal accomplishment (PA) is a positive symptom and reflects feelings of capability and job satisfaction.

Items were rated on a 7-point frequency scale and assigned 0–6 points, such that more points indicated a higher propensity for having burn out. Cut-off scores were provided by the Dutch Central Bureau of Statistics (www.cbs.nl, http://www.tno.nl/downloads/Rapport_NEA_20101.pdf).

Respondents provided demographic information on gender, age, marital status, children living at home; type of specialty; years of professorship, and main professional activity (research, education, patient care or management).

### Statistical Analysis

Independence of bivariate association was assessed by multiple linear regression analysis using the stepwise forward method. Pearson’s correlation coefficients were calculated to examine relationships between publication pressure and burn out scores.

An age-adjusted General Additive Model (GAM)-curve was constructed to graphically display the association between publication pressure and specific burn put scores [25], using the statistical software package R, version 2.15.1 (R Development Core Team, R foundation, USA).

## Results

In total, we used 1366 e-mail addresses. Of these, 160 bounced, most often because the addresses no longer existed, or provided an out-of-office reply. Of the 1206 professors left; 578 responded (49%), of whom 437 (36%) completed the full questionnaire. The demographic data of the complete responders are summarized in table 2.

**Table 2:** Demographics.

Demographics			
		N = 437	%
Gender	Male	345	79
	Female	92	21
Age	26–35	1	0,2
	36–45	35	8,0
	46–55	20	47,1
	56–65	190	43,5
	65 and older	5	1,1
Marital status	Married or cohabiting	401	92
	Single	36	8
Home livingchildren	None	217	50
	1	56	13
	2	96	22
	3 or more	68	15
Years of professorship	0–5	150	34
	6–10	129	30
	11–15	86	20
	15 or more	72	16
Nr. 1 Work priority	Research	255	59
	Education	40	9
	Patient care	63	14
	Management	79	18
Appointment	Temporary	144	33
	Permanent	293	67

### Publication Pressure Questionnaire (PPQ)

Table 1 lists the questions and responses. The responses to some of the key questions indicate that the majority (54%, item 13) rates publication pressure as 'excessive'. In addition, 1 out of every 3 to 4 respondents (items 1,10 and 12) believes that the pressure to publish has detrimental effects on the validity and credibility of medical science. Finally, 24% (item 24) qualifies publication pressure as having a ‘sickening’ effect on medical science.

We use the sum score of the 14 items (marked as **bold** in table 1) of the condensed, validated PPQ for further analysis of correlations and determinants.

### Correlates of Publication Pressure

In univariate analyses, female gender and having home living children were positively, and the number of years of professorship was negatively associated with publication pressure (table 3, top).

**Table 3:** Univariate and Multivariate analysis comparing independent variables with the PPQ.

Univariate	Beta	CI 95% lower bound	CI 95% upper bound	P value
Age (10 year)	−0.623	−1.75	0.51	0.28
Gender (female)	1.851	0.04	3.89	0.05
Marital status (single)	−1.421	−4.12	1.78	0.30
Homeliving children (increase of 1 child)	0.718	0.08	1.36	0.03
Fixed position (yes)	−1.116	−2.69	0.46	0.17
Years prof (increase of 5 years)	−1.013	−1.69	−0.33	0.004
Multivariate				
Gender (female)	1.528	−0.30	3.36	0.10
Homeliving children	0.479	0.17	−0.20	0.17
Years of professorship	−0.739	−1.48	−0.003	0.05

Multivariate analysis identified the number of years of professorship as the single independent (inverse) determinant of publication pressure. The univariate effects of gender and having home living children were reduced to non-significant trends (table 3, bottom).

### Prevalence of Burn Out and Association with Publication Pressure Questionnaire

Of all respondents, 24% met the formal Dutch CBS criteria for having burn out. Mean scores of EE, DP and PA were 11.9 (interquartile range (IQR) 5–16), 4.4 (IQR 1–6) and 30.9 (IQR 28–35).

The PPQ-score was significantly associated with scores on all 3 subscales of the burnout questionnaire. Correlations were strongest for the emotional exhaustion subscale (Pearson’s correlation coefficient 0.45, p<0.001). The cumulative publication pressure score showed a weaker, but highly significant (inverse) correlation with depersonalisation and personal accomplishment (Pearson’s correlation coefficient 0.29 and −0.15 respectively, p<0.001).

**Figure 1:**
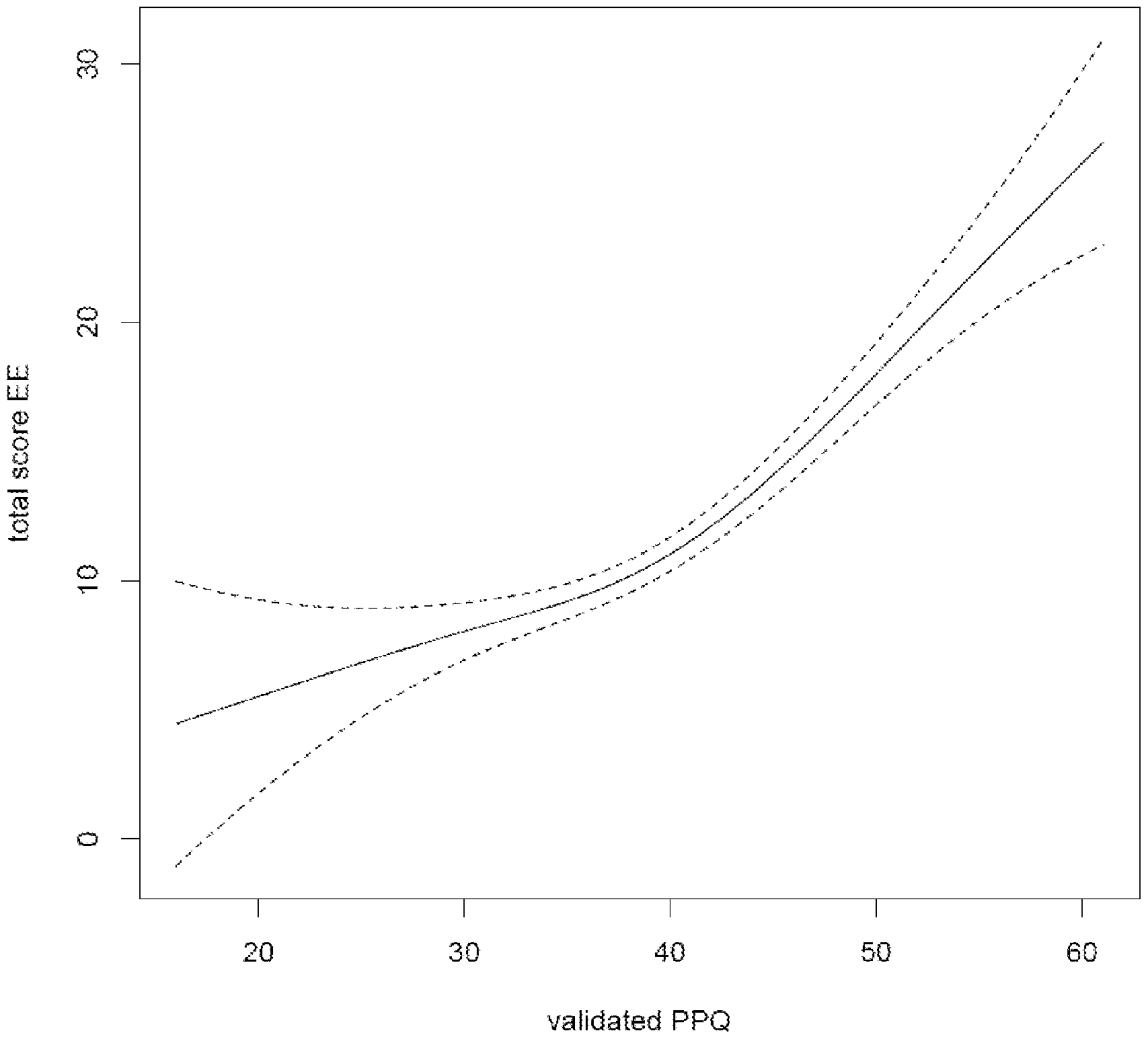
General Additive Model-curve, demonstrating the association between the sum score on the validated 14-item publication pressure questionnaire, and the Emotional Exhaustion component of the burn out index, adjusted for gender to reduce residual confounding (Benedetti A and Abrahamowicz M). The dotted line represents the 95% Confidence interval.

Figure 1 illustrates the gender-adjusted correlation between the PPQ-score and the emotional exhaustion subscale of the burnout questionnaire.

## Discussion

Our study suggests that a substantial proportion of the responding medical professors judge publication pressure as having become excessive, and a substantial part believes that this affects the validity and credibility of medical science. Furthermore, they personally experience publication pressure, and believe the publication pressure negatively influences their work both in science as well as in other academic tasks, such as clinical and educational work. There is a high level of burn out among medical professors in The Netherlands, and publication pressure correlates positively with burn out symptoms, particularly in the domain of emotional exhaustion.

Our findings are generally in line with the few studies published on this subject, which relate publication pressure to publication bias [26], and report high personal pressure associated with academic competition [12]. Our results add to these previous studies in that our survey is nationwide, identify possible determinants of experienced publication pressure, and address associations of such pressure with perception and trust in medical science. Finally, we addressed personal consequences of publication pressure in terms of burn-out symptoms.

The causes of publication pressure are important to consider. Although our study was not specifically designed to identify such causes, the responses to statements #4, 5 and 15 in the questionnaire suggest that increasing emphasis of quantitative aspects of scientific output plays an important role. This contention is shared by others, who have published on the effects of introduction of the Hirsch-index on the medical scientific field [12]; [27], [28].

Another important cause may be the importance of bibliographic parameters in the assignment of research funding, grants, scholarships and academic positions. In this area, ambition and prestige is partly built on bibliographic parameters, potentially compromising the impact of clinical and educational performance.

Publication pressure may have adverse effects on medical science. In the respondents’ opinion, publication pressure can adversely affect validity and reliability of the medical literature. A bias towards positive outcomes has indeed been suggested in increasingly competitive, academic environments [26]. Competitiveness and precariousness of scientific careers have increased, and evidence that this might contribute to scientific bias and even misconduct has accumulated [29]. Scientists in focus groups suggested that the need to compete in academia is a threat to scientific integrity [12]. Those found guilty of scientific misconduct often invoke excessive pressure as part of the explanation for their actions [12], [27], [30], [31]. Surveys suggest that competitive research environments decrease the likelihood to follow scientific ideals and increase the likelihood to witness scientific misconduct [27], [32] Another potential adverse effect is neglect of other important academic skills, such as education and patient care.

Apart from general effects on medical science and practice, our study suggests that excessive publication pressure has detrimental effects on personal well-being. A large proportion of the respondents experiences publication pressure as a burden. Moreover, our study indicates that publication pressure causes them to develop a cynical view on medical science, and may be associated with increased risk of developing burn-out.

The central Bureau of Statistics Netherlands has reported that 8–11% of the Dutch working population is burned out (www.cbs.nl, http://www.tno.nl/downloads/Rapport%20NEA%2020101.pdf). This suggests that burn out is more common among medical professors (24%), which is in line with previous reports [16]–[18], [20]. Burn out among medical professionals not only causes personal suffering, but also leads to decreased work performance and jeopardises the quality of patient care [33], [34].

A number of limitations of our study needs to be addressed. Firstly, with a response rate of 36%, we cannot rule out response bias. Nevertheless, this response rate of 36% is normal for internet-based surveys in the general population [35] and for similar surveys among academics [36] http://www.supersurvey.com/papers/supersurvey_white_paper_response_rates.pdf).

Although the response rate of 36% could be considered average and ideally would have been higher, it does represent a sample of the total population of medical professors in the Netherlands, were we reached all medical professors, not of a sample of them.

Regardless of whether a higher response rate would have been theoretically feasible, it is prudent to discuss the direction of potential sources of response bias. We think that response bias in our study may have been bidirectional; non-response may be related to lack of time or sense of task overload. This is demonstrated by Prins and colleagues [19] who sent out an ultra brief questionnaire to all the nonresponding residents asking for reasons of lack of cooperation. 23% of non-respondents did not participate because of lack of time and 11% did not because of lack of energy. Similar reasons for non-response in our survey may have caused underestimation of burn-out symptoms and of discontent with publication pressure. Such underestimation might also have resulted from a taboo on personal pressure and burn out, causing respondents to downplay the severity and personal impact of publication pressure, despite guaranteed anonymity for the respondents.

On the other hand, lack of cooperation may be related to the subject of the survey. Possibly, some medical professors consider publication pressure irrelevant, and thus refuse to participate. Such bias would conceivably have caused overestimation of burn-out and disapproval of publication pressure among respondents. Another potential source of overestimation of the problem is framing. The invitation e-mail did not contain words as ‘burn out’ and 'publication pressure' but was phrased using more neutral words as 'work engagement' and 'publication culture', to increase response rate and avoid framing and related response bias as much as possible. Some of the statements we used for the questionnaire were perhaps 'framed', but we believe questionnaire statements should connect to the context of the ongoing academic debate on the subject of publication pressure, which is framed in itself. We therefore chose to include provocative 'negative' statements/questions, but the dominance of such negative statements persuaded us to mix them with more positively framed statements/questions. The 'negative' questions/statements connect to the current public and academic debate, and are expected to be recognised as such. The 'positive' ones are those that provoke respondents to take the opposite, or at least a more reflective, position. Inclusion of such inversed ’positive’ statements improves psychometric properties of the questionnaire and downplays yeah-saying [22] (see methods).

Burn out was measured using the Maslach Burnout Inventory (MBI). This inventory has its advantages and shortcomings. The MBI is regularly used in similar types of studies addressing work-related psychological stress in medical professionals. Burn-out is not a specific, but in our view a conceivable consequence of publication pressure, which is indeed supported by the literature [13]. For further research, the use of alternative job-stress questionnaires or surveys for measuring psychiatric symptoms should be considered.

Another potential source of bias, in either direction, could be the use of an online questionnaire. The validity of online questionnaires has, however, been extensively studied and there is no evidence that web based questionnaires are less valid than ‘live’ questionnaires [37].

Finally, the timing of the study (September–October 2011) could have influenced the results and possibly attenuate burn out symptom scores, since national holidays are held in July and August, and academic work normally starts in the beginning of September.

We chose to include full medical professors who are in various ways the leading authorities in their research field and have the key positions in the hierarchy in Dutch academia in practising, steering and teaching science. They are role models for younger scientists in the Dutch medical Universities. Therefore, their opinion on publication culture is of main importance. Also, this particular group of medical professionals was not previously examined in a similar way. We can only speculate how other physicians or researchers in academic medical centres, would have responded to the questionnaires. Pressure could be worse among individuals from lower hierarchical groups, who need scientific output to boost their careers, which may predispose them to experience pressure and stress related symptoms [19]. Furthermore, our data suggests that accumulation of years of professorship is an important determinant for lower publication pressure scores. Is this light, it is conceivable that being in the early years of academic career development as such is associated with higher levels of publication pressure.

In conclusion, there is a high level of experienced publication pressure in medical professors. No less than 24% of them meet the criteria for having a burn-out, which is statistically correlated to reported publication pressure. These results shed a dim light on some inner thoughts and feelings of our academics leaders in science, education and patient care. Further research is obviously needed, but actions to address the upcoming ‘more is better’ culture in medical science already appear necessary.
